# Abnormal Degree Centrality in White Matter Hyperintensities: A Resting-State Functional Magnetic Resonance Imaging Study

**DOI:** 10.3389/fpsyt.2021.684553

**Published:** 2021-07-13

**Authors:** Baogen Du, Shanshan Cao, Yuanyuan Liu, Qiang Wei, Jun Zhang, Chen Chen, Xiaojing Wang, Yuting Mo, Jiajia Nie, Bensheng Qiu, Panpan Hu, Kai Wang

**Affiliations:** ^1^Department of Neurology, The First Affiliated Hospital of Anhui Medical University, Hefei, China; ^2^Anhui Province Key Laboratory of Cognition and Neuropsychiatric Disorders, Hefei, China; ^3^Collaborative Innovation Center of Neuropsychiatric Disorders and Mental Health, Hefei, China; ^4^Department of Neurology, The Second Affiliated Hospital of Anhui Medical University, Hefei, China; ^5^Hefei National Lab for Physical Sciences at the Microscale and the Centers for Biomedical Engneering, University of Science and Technology of China, Hefei, China; ^6^Hefei Comprehensive National Science Center, Institute of Artificial Intelligence, Hefei, China; ^7^The School of Mental Health and Psychological Sciences, Anhui Medical University, Hefei, China

**Keywords:** degree centrality, cerebral small vessel disease, white matter hyperintensites, cognitive impairment, functional connectivity

## Abstract

**Background:** White matter hyperintensities (WMHs) are a common occurrence with aging and are associated with cognitive impairment. However, the neurobiological mechanisms of WMHs remain poorly understood. Functional magnetic resonance imaging (fMRI) is a prominent tool that helps in non-invasive examinations and is increasingly used to diagnose neuropsychiatric diseases. Degree centrality (DC) is a common and reliable index in fMRI, which counts the number of direct connections for a given voxel in a network and reflects the functional connectivity within brain networks. We explored the underlying mechanism of cognitive impairment in WMHs from the perspective of DC.

**Methods:** A total of 104 patients with WMHs and 37 matched healthy controls (HCs) were enrolled in the current study. All participants underwent individual and overall cognitive function tests and resting-state fMRI (rs-fMRI). WMHs were divided into three groups (39 mild WMHs, 37 moderate WMHs, and 28 severe WMHs) according to their Fazekas scores, and the abnormal DC values in the WMHs and HCs groups were analyzed.

**Results:** There was a significant difference in the right inferior frontal orbital gyrus and left superior parietal gyrus between the WMHs and HCs groups. The functional connectivity between the right inferior frontal orbital gyrus and left inferior temporal gyrus, left superior parietal gyrus, and left parietal inferior gyrus was also different in the WMHs group.

**Conclusion:** The change in DC value may be one of the underlying mechanisms of cognitive impairment in individuals with WMHs, which provides us with a new approach to delaying cognitive impairment in WMHs.

## Introduction

Cerebral small vessel disease (CSVD) is a common cerebrovascular disease that refers to a syndrome of clinical and imaging findings (1). The features seen on neuroimaging include white matter hyperintensities (WMHs), lacunar infarcts, recent small subcortical infarcts, perivascular spaces, microbleeds, and brain atrophy (2); the most common neuroimaging feature is WMHs, which are often considered to primarily represent ischemic damage caused by CSVD (3, 4). WMHs have been detected in 77.8% of healthy elderly individuals between 60 and 82 years of age (5). These WMHs are among the most important vascular contributors to cognitive decline, dementia, and parkinsonism (6). Cerebral small vessel disease (CSVD) is a common disease in elderly and has long been implicated with cognitive impairment, dementia, and stroke, causing up to 45% of dementia cases worldwide, and accounting for approximately one-quarter of all strokes (3). A longitudinal study involved 818 individuals from the Alzheimer Disease Neuroimaging Initiative (ADNI)-2 dataset from August 2010 to May 2017 suggested that WMHs had a significant impact on cognitive impairment and elevate the risk of conversion to dementia. They even pointed that WMHs volume can be regarded as a non-invasive marker of cognitive degeneration (7). A systematic review and meta-analysis of 36 prospective studies to explore the association between white matter hyperintensities and risks of cognitive impairment and dementia indicated that WMHs were associated with increased risk of cognitive dysfunction (8). The previous studies found an association of progression of white matter hyperintensities with a faster decline of executive functions or processing speed (9–12). Another study indicated that WMHs volume were associated with an increased rate of decline in global cognition, perceptual speed, working memory, episodic memory, and semantic memory. Associations persisted after adjustment for total gray matter volume, vascular risk factors, and vascular diseases (13).

Functional magnetic resonance imaging (fMRI) is an outstanding tool that helps with non-invasive examinations. Using resting-state fMRI (rs-fMRI), researchers have found that the brain exhibits consistent low frequency fluctuations in the 0.01–0.08 Hz range, and that these frequencies could be used to indicated intrinsic activity within the whole brain (14). rs-fMRI techniques have been increasingly utilized to investigate functional alterations related to WMHs. It has been reported that changes in the structural connectivity of brain networks have been found in WMHs, and abnormalities of the structural connectivity to central network nodes may lead to the occurrence of cognitive impairment related to WMHs to some extent (15, 16). Therefore, a method to explore the disruption of functional connectivity in WMHs is needed. However, the magnetic resonance technology of previous studies tends to focus on the region of interest (ROI) (17–20). In previous study, thalamus was used as ROI to study the changes of functional connectivity (FC) of thalamic cortex in WMHs, and they found that abnormal thalamocortical FC was closely related with cognitive impairments in WMHs (21). Similarly, some people pay their attention to the relationship between white matter hyperintensity in basal ganglia and cognitive function, they found that White Matter Hyperintensities Relate to Basal Ganglia Functional Connectivity and Memory Performance in amnestic mild cognitive impairment (MCI) and subcortical vascular MCI (22). With the rapid development of neuroimaging techniques, an increasing number of imaging techniques are used in the diagnosis of neurological diseases. Degree centrality (DC) analysis is one technique that attracted our attention. Degree centrality is a particularly interesting graph metric of graph based network analysis methods (23). It does not depend on prior assumptions as the choice of seed region or a predefined template to account for the brain regions. It is a robust index of focal connectivity ascertained by counting the number of direct connections from one node to all other nodes (23, 24). Fair to good test-retest reliability of this technique has been proved in previous studies (25–27). Unlike seed-based approaches or independent component analysis, centrality measures take into account a given region's relationship with the entire functional connectome and not just its relation to individual regions or to separate larger components (28). DC is the most direct measure of node centrality in network analysis. Generally speaking, a higher DC value indicates a more important node. Currently, DC has been used to evaluate changes in the brain network in diseases such as depression (29), schizophrenia (30), asthma (31), and Parkinson's disease (32). WMHs are mainly manifested as abnormal signals in white matter. Structural white matter connectivity is the basis of functional connectivity. We speculated that WMHs can affect white matter connectivity and further cause abnormal functional connectivity of cortex and subcortical nuclei. To investigate the mechanisms underlying cognitive impairment, we used fMRI to analyze the DC value in patients with WMHs and HCs. In the present study, we analyzed differences in the DC value between patients with WMHs and HCs to further explore its possible mechanism.

## Materials and Methods

### Participants

We recruited 104 patients with WMHs (39 with mild WMHs, 37 with moderate WMHs, and 28 with severe WMHs) and 37 HCs. The clinical manifestations of WMHs are heterogeneous, and the clinical symptoms of WMHs do not exhibit a pattern; therefore, there are no established diagnostic criteria. Consequently, the diagnostic criteria mainly depend on imaging features. As a result, the inclusion criteria for the WMHs group were as follows: (1) age between 40 and 80 years and (2) visible WMHs on T2 fluid-attenuated inversion recovery (T2 FLAIR). The exclusion criteria were as follows: (1) intracranial and extracranial stenosis >50%; (2) Trial of Org 10172 in Acute Stroke Treatment classification suggestive of cardiogenic stroke; (3) non-CSVD-related WMHs (e.g., multiple sclerosis); (4) mental disorders or alcohol addiction; (5) dementia or tumors; (6) intracranial hemorrhage; (7) significant hearing or visual impairment, physical movement disorders that prevented co-operation during cognitive testing, (8) language barrier, and (9) contraindications to MRI or known claustrophobia. Patients with WMHs were classified based on the Fazekas scores as mild group, moderate group, or severe group. Periventricular hyperintensity (PVH) and deep white matter hyperintensity (DWMH) were scored separately using a four-point scale according to the Fazekas scale on FLAIR images. The PVH scores were categorized as follows: 0, absent; 1, caps or pencil-thin lining around ventricles; 2: smooth halo around ventricles; 3: irregular PVH lesions extending into the deep white matter. DWMH scores were categorized as follows: 0, absence; 1, punctate foci; 2, beginning confluence of foci; and 3, large confluent areas. The Fazekas score is the sum of the PVH and DWMH scores. The mild WMHs group scored 1–2, the moderate WMHs group scored 3–4, and the severe WMHs group scored 5–6. Finally, we included 39 patients in the mild group, 37 patients in the moderate group, and 28 patients in the severe group. Thirty-seven HCs who were relatives of patients with WMHs and social recruits studied during the same period who matched the demographic data of the patients with WMHs, including age, sex, and years of education, and who had no previous history of neurological disease or mental illness were also included. Imaging revealed no WMHs.

All participants provided written informed consent to our protocol, which was approved by the medical ethics committee of the First Affiliated Hospital of Anhui Medical University.

### Study Design and Process

The screening and examination for WMHs was performed by trained neurologists and graduate students in neurology. We divided patients into three grades (mild, moderate, and severe) according to the Fazekas Scale, after which we collected the rs-fMRI and cognitive assessment data for WMHs, and finally completed the statistical analysis of relevant data.

### Cognitive Examination

A cognitive examination was completed by all participants on the same day as the MRI scan, cognitive examination includes both overall and single cognitive tests. We used the Montreal Cognitive Assessment (MoCA) to evaluate the overall cognitive function of participants and the Generalized Anxiety Disorder 7 (GAD-7) Scale, Patient Health Questionnaire 9 (PHQ-9), Trail Making Test (TMT-A and TMT-B), Boston Naming Test (BNT), and auditory verbal learning test (AVLT) were used to evaluate cognitive function, respectively.

### MRI Data Acquisition

All participants completed fMRI in the magnetic resonance chamber at the Information Science Center of the University of Science and Technology of China. During the scan, all participants were told to lie flat in the machine while resting with their eyes closed, but not to fall asleep, not to move their bodies, and not to think about anything.

Functional imaging was conducted using a 3.0-T MRI scanner (Discovery GE750w; GE Healthcare, Buckinghamshire, UK), each participant underwent MRI scanning, and the functional imaging was composed of 217 echo-planar imaging volumes. The specific parameters were as follows: Time of Repetition (TR) = 2,400 ms, Time of Echo (TE) = 30 ms, flip angle = 90°, matrix size = 64 × 64, field of view = 192 × 192 mm^2^, slice thickness = 3 mm, no gap, and 46 continuous slices (voxel size = 3 × 3 × 3 mm^3^). T1-weighted anatomic images with 188 slices were also acquired in the sagittal orientation (TR = 8.16 ms; TE = 3.18 ms; flip angle = 12°; field of view = 256 × 256 mm^2^; slice thickness = 1 mm; and voxel size = 1 × 1 × 1 mm^3^).

### Data Pre-processing

Functional data were preprocessed using the Data Processing Assistant of the Resting-State Functional MR Imaging toolkit (DPARSF) (http://www.fil.ion.ucl.ac.uk/spm) (33) and a software package based on Statistical Parametric Mapping software (SPM12, http://www.fil.ion.ucl.ac.uk/spm). All the data were processed with the following steps: (1) conversion of data format; (2) discarding of the first 10 volumes to exclude the influence of unstable longitudinal magnetization; (3) slice-timing correction; (4) motion correction; (5) normalization and registration to the Montreal Neurological Institute (MNI) template space; and (6) spatial smoothing based on the unified segmentation of structural images (Gaussian kernel, full width at half-maximum = 8 mm) and motion correction by scrubbing; (7) Filter (0.01–0.1 Hz). All participants showed a maximum displacement of <3 mm and an angular motion of <3° and were thus included in the subsequent analyses.

### DC Calculation

The DC value is used to evaluate DC. Degree centrality represents the number of direct connections for a given voxel in the voxel-based graphs. In the present study, the preprocessed fMRI data were used to compute the voxel-based whole-brain correlation analysis (34). The time course of each voxel in each brain was correlated to every other voxel time course in the gray matter (GM) mask. Thus, we could acquire an n × n matrix of Pearson's correlation coefficients between any pair of voxels, where n is the voxel number of the GM mask. Next, we transformed the Pearson's correlation data into normally distributed Fisher Z-scores and constructed the whole-brain functional network by thresholding each correlation at *r* > 0.25 (24). The threshold was the default setting while constructing the DC map. Only positive Pearson's correlation coefficients were considered in the present study. For a given voxel, the DC was calculated as the sum of the significant connections at the individual level.

### Statistical Analysis

The differences in age, years of education and cognitive tests were assessed with one-way ANOVA analysis, and sex distribution, diabetes, hyperlipidemia, drinking history, and smoking history between the HCs and WMHs groups were assessed with the chi-square test using the Statistical Package for the Social Sciences 23.0 (SPSS, Chicago, IL, United States). The Least—Significant Difference was used for *post-hoc* analysis (significant for *P* < 0.05). We used DPARSF to extract the average DC and FC values of the brain region with significant difference, and then introduced it into SPSS23.0, and we used one-way ANOVA to analyzed the differences among groups. The statistical significance threshold for DC and FC was set to *P* < 0.001 at the voxel level and *P* < 0.05 at the cluster level with Gaussian random field (GRF) correction. Meanwhile, the Pearson's correlation analysis was conducted to find out the relationship between abnormal DC value in the related brain area and cognitive function, we only analyzed the correlation between the DC values of brain regions with differences among groups and the cognitive tests with differences among groups.

## Results

### Demographics, Cognitive Examination, and Clinical Characteristics

There were no significant differences in age, years of education, or sex distribution between the HCs and WMHs groups. The proportion of patients with hypertension was lower in the HCs groups than in the WMHs group, but there were no significant differences among the four groups in terms of diabetes, hyperlipidemia, smoking history, and drinking history. Demographic information and WMHs marker information are shown in Table 1. Regarding the severity of WMHs, the quantitative assessment method we used was in good agreement with the Fazekas score (*P* < 0.001, *r* = 0.499). The distribution of lacunae and microbleed lesions is shown in Table 2. The neuropsychological test results of the participants in the four groups are shown in Table 3. However, a few participants did not complete the ESWAN sequence scan (HCs group, 5 participants; mild WMHs group, 13 patients; moderate WMHs group, 7 patients; severe WMHs group, 7 patients).

**Table 1:** Demographic and WMHs neuroimaging manifestations of participants in the four groups [Mean (SD)].

	**HCs** ** (*n* = 37)**	**Mild WMHs** ** (*n* = 39)**	**Moderate WMHs** ** (*n* = 37)**	**Severe WMHs** ** (*n* = 28)**	***F***	**χ^**2**^**	***P***
Age (years)	60.65 (5.91)	63.77 (8.23)	65.08 (10.03)	65.04 (6.90)	2.412		0.069
Education (years)	9.05 (3.61)	8.41 (4.16)	8.00 (3.84)	7.75 (3.88)	0.732		0.535
Female	19	16	14	16		3.205	0.361
Hyperlipidemia	8	10	9	8		7.060	0.070
Hypertension	8	21	22	21		20.419	<0.001^***^
Diabetes	2	8	7	4		4.081	0.253
Drinking history	12	9	16	12		4.420	0.220
Smoking history	9	18	15	11		4.166	0.244
Microbleeds	0.33 (0.60) ^c^,	2.26 (3.61)^e^	1.50 (2.35)^f^	6.29 (14.97)^c^^,^ ^e^^,^ ^f^	3.429		0.020^**^
Lacunes	0.00 (0.00)^a^^,^ ^b^^,^ ^c^	0.51 (0.91)^a^^,^ ^d^	0.78 (1.25)^b, d^	0.64 (1.06) ^c^	4.782		0.003^**^
WMHs volume	/	11457.35 (12493.05)^d^^,^ ^e^	18067.56 (10873.84)^d^^,^ ^f^	30658.380 (12033.91)^e^^,^ ^f^	21.696		<0.001^***^
Fazekas	0.00 (0.00)^a^^,^ ^b^^,^ ^c^	1.64 (0.49)^a^^,^ ^d^^,^ ^e^	3.59 (0.50)^b^^,^ ^d^^,^ ^f^	5.43 (0.50)^c^^,^ ^e^^,^ ^f^	1004.49		<0.001^***^

a *Healthy control group vs. mild WMHs group significantly different (P < 0.05)*,

b *Healthy control group vs. moderate WMHs group significantly different (P < 0.05)*,

c *Healthy control group vs. severe WMHs group significantly different (P < 0.05)*,

d *Mild WMHs group vs. moderate WMHs group significantly different (P < 0.05)*,

e *Mild WMHs group vs. severe WMHs group significantly different (P < 0.05)*,

f *Moderate WMHs group vs. severe WMHs group significantly different (P < 0.05)*.

*SD, standard deviation*.

*Volume are in cubic millimeters*.

*** *Significant at 0.001 level and*

** *Significant at 0.01 level (2-tailed)*.

**Table 2:** Distribution of neuroimaging manifestations in HCs and WMHs [Mean (SD)].

	**Microbleeds**				**Lacunes**			
	**HCs** ** (*n* = 32)**	**Mild WMHs** ** (*n* = 26)**	**Moderate WMHs** ** (*n* = 30)**	**Severe WMHs** ** (*n* = 21)**	**HCs** ** (*n* = 37)**	**Mild WMHs** ** (*n* = 39)**	**Moderate WMHs** ** (*n* = 37)**	**Severe WMHs** ** (*n* = 28)**
**Infratentorial**									
Cerebellum	0.03 (0.18)	0.08 (0.27)	0.13 (0.57)	0.33 (0.80)	0.00 (0.00)	0.12 (0.33)	0.13 (0.35)	0.86 (3.50)
Pons	0.09 (0.30)	0.23 (0.71)	0.10 (0.31)	0.33 (0.35)	0.00 (0.00)	0.15 (0.37)	0.33 (0.18)	0.14 (0.48)
Mesencephalon	0.03 (0.18)	0.08 (0.27)	0.07 (0.25)	0.57 (1.36)	0.00 (0.00)	0.15 (0.46)	0.33 (0.18)	0.14 (0.65)
Any	0.03 (0.18)	0.42 (1.17)	0.37 (0.93)	1.25 (3.02)	0.00 (0.00)	0.04 (0.20)	0.33 (0.18)	0.29 (1.10)
**Deep**									
Basal ganglia	0.00 (0.00)	0.04 (0.20)	0.00 (0.00)	0.19 (0.87)	0.00 (0.00)	0.23 (0.51)	0.17 (0.38)	0.68 (1.29)
Thalamus	0.03 (0.18)	0.23 (0.51)	0.17 (0.38)	0.68 (1.29)	0.00 (0.00)	0.08 (0.27)	0.17 (0.46)	0.43 (1.33)
Internal capsule	0.00 (0.00)	0.08 (0.27)	0.17 (0.46)	0.43 (1.33)	0.00 (0.00)	0.46 (0.86)	0.33 (0.92)	0.62 (1.72)
Any deep	0.00 (0.00)	0.46 (0.86)	0.33 (0.92)	0.62 (1.72)	0.00 (0.00)	0.12 (0.33)	0.20 (0.66)	0.24 (0.89)
**Subcortical**									
Frontal	0.03 (0.18)	0.12 (0.33)	0.20 (0.66)	0.24 (0.89)	0.00 (0.00)	0.08 (0.27)	0.13 (0.57)	0.33 (0.80)
Parietal	0.03 (0.18)	0.12 (0.33)	0.13 (0.35)	0.86 (3.50)	0.00 (0.00)	0.23 (0.71)	0.10 (0.31)	0.33 (0.35)
Occiptal	0.00 (0.00)	0.15 (0.37)	0.33 (0.18)	0.14 (0.48)	0.00 (0.00)	0.08 (0.27)	0.07 (0.25)	0.57 (1.36)
Temporal	0.03 (0.18)	0.15 (0.46)	0.33 (0.18)	0.14 (0.65)	0.08 (0.27)	0.42 (1.17)	0.37 (0.93)	1.25 (3.02)
Any subcortical	0.00 (0.00)	0.04 (0.20)	0.33 (0.18)	0.29 (1.10)	0.00 (0.00)	0.04 (0.20)	0.00 (0.00)	0.19 (0.87)
Any	0.31 (0.59)	2.19 (3.58)	1.73 (2.63)	6.33 (14.96)	0.00 (0.00)	2.19 (3.58)	1.73 (2.63)	6.33 (14.96)

*SD, standard deviation; WMHs, white matter hyperintensities*.

**Table 3:** Neuropsychological tests of participants in the four groups [Mean (SD)].

	**HCs** ** (*n* = 37)**	**Mild WMHs** ** (*n* = 39)**	**Moderate WMHs** ** (*n* = 37)**	**Severe WMHs** ** (*n* = 28)**	***F***	***P***
MoCA	22.26 (3.00)^c^	21.16 (4.32)^d^	20.45 (4.43)^e^	17.48 (5.06)^c^^,^ ^d^^,^ ^e^	6.681	<0.001^***^
GAD-7	2.42 (3.29)	2.61 (4.10)	2.68 (3.34)	4.88 (5.34)	2.765	0.068
PHQ-9	3.61 (4.90)	3.74 (4.56)	5.10 (4.58)	6.75 (5.81)	3.018	0.062
TMT-A	63.05 (23.83)^a^^,^ ^b^^,^ ^c^	82.20 (37.31)^a^^,^ ^d^	81.71 (34.36)^b^^,^ ^e^	107.26 (37.74)^c^^,^ ^d^^,^ ^e^	7.759	<0.001^***^
TMT-B	132.61 (43.98)^c^	156.82 (74.22)^d^	162.22 (74.47)^e^	208.21 (81.54)^c^^,^ ^d^^,^ ^e^	6.285	0.001^**^
BNT	13.80 (1.74)^a^^,^ ^b^^,^ ^c^	12.89 (1.54)^a^	13.08 (1.68)^b^	12.76 (1.35)^c^	4.797	0.003^**^
AVLT-study	8.04 (1.71)^c^	7.63 (2.24)^d^	7.31 (1.52)	6.55 (2.00)^c^^,^ ^d^	3.220	0.025^*^
AVLT-immediate	7.97 (1.68)^c^	7.63 (2.24)^d^	7.18 (1.78)^e^	6.39 (1.93)^c^^,^ ^d^^,^ ^e^	4.942	0.003^**^
AVLT-delay	8.82 (2.61)^b^^,^ ^c^	7.95 (3.76)	7.14 (2.88)^b^	5.89 (3.34)^c^	5.053	0.022^*^
AVLT-recognition	13.55 (1.43)^c^	13.53 (3.68)^d^	12.81 (2.73)^e^	11.29 (3.41)^c^^,^ ^d^^,^ ^e^	4.734	0.008^*^

a *Healthy control group vs. mild WMHs group significantly different (P < 0.05)*,

b *Healthy control group vs. moderate WMHs group significantly different (P < 0.05)*,

c *Healthy control group vs. severe WMHs group significantly different (P < 0.05)*,

d *Mild WMHs group vs. severe WMHs group significantly different (P < 0.05), and*

e *Moderate WMHs group vs. severe WMHs group significantly different (P < 0.05)*.

*SD, standard deviation; MoCA, Montreal Cognitive Assessment; PHQ, Patient Health Questionnaire; GAD, Generalized Anxiety Disorder; AVLT, Chinese Auditory Learning Test; TMT, Trial Making Test; BNT, Boston Naming Test*.

*** *Significant at 0.001 level*,

** *significant at 0.01 level, and*

* *significant at 0.05 level (2-tailed)*.

### DC Analysis

**Figure 1:**
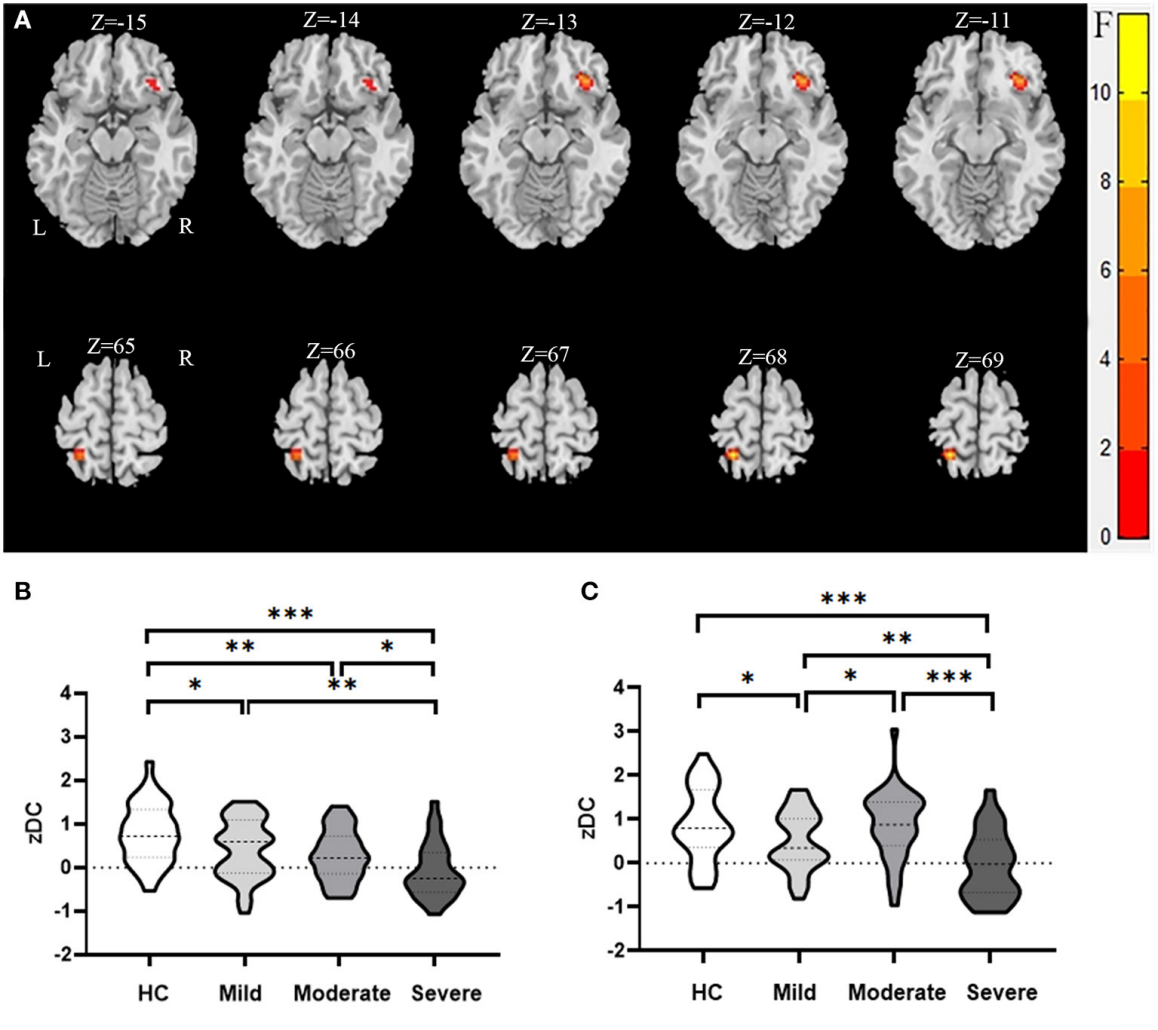
Brain regions showing abnormal DC value in the WMHs compared to the HCs in MNI space. **(A)** Significant DC value differences were observed in the right inferior frontal orbital gyrus and the left superior parietal gyrus [−15, −14, −13, −12, −11, 65, 66, 67, 68, 69 in **(A)** are the coordinates of Z-axis in Montreal Neurological Institute space, and the unit of coordinate system is millimeter]. **(B,C)** Mean values of altered DC values in the right inferior frontal orbital gyrus and the left superior parietal gyrus between the HCs and WMHs groups. ***Significant at 0.001 level, **significant at 0.01 level, and *significant at 0.05 level (2-tailed).

The one-way analysis of variance revealed significantly different DC values within the right inferior frontal orbital gyrus and the left superior parietal gyrus in the WMHs groups as compared to those in the HCs group (*P* < 0.05, cluster; *P* < 0.001, voxel; GRF) (Table 4, Figure 1).

**Table 4:** Brain regions showing differences in the degree centrality between the HCs group and the WMHs group.

**Brain regions**	**Peak MNI**	**Number of voxels**	***F*-value**	***P* (GRF)**
	**X Y Z**			
Right inferior frontal orbital gyrus	30 33 −12	25	9.3406	Cluster <0.05, voxel <0.001
Left superior parietal gyrus	−24 −48 69	25	11.8109	Cluster <0.05, voxel <0.001

*MNI, Montreal neurological institute; WMHs, white matter hyperintensities*.

### Functional Connectivity Analysis

**Figure 2:**
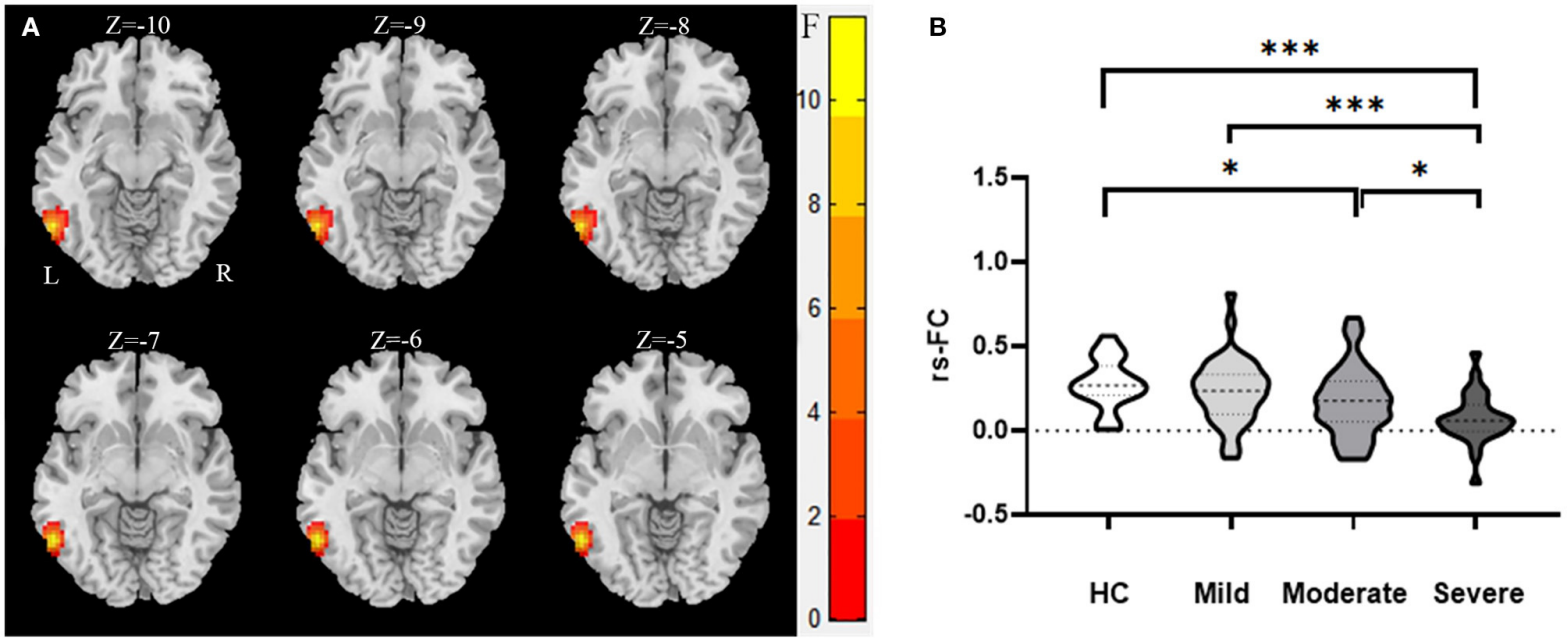
The abnormal functional connectivity between the right inferior frontal orbital gyrus and left inferior temporal gyrus of the WMHs in MNI space. **(A)** Significant abnormal functional connectivities were observed between the right inferior frontal orbital gyrus and left inferior temporal gyrus [−10, −9, −8, −7, −6, −5 in **(A)** are the coordinates of Z-axis in Montreal Neurological Institute space, and the unit of coordinate system is millimeter]. **(B)** Mean values of the abnormal functional connectivities in these groups. ***Significant at 0.001 level and *significant at 0.05 level (2-tailed).

**Figure 3:**
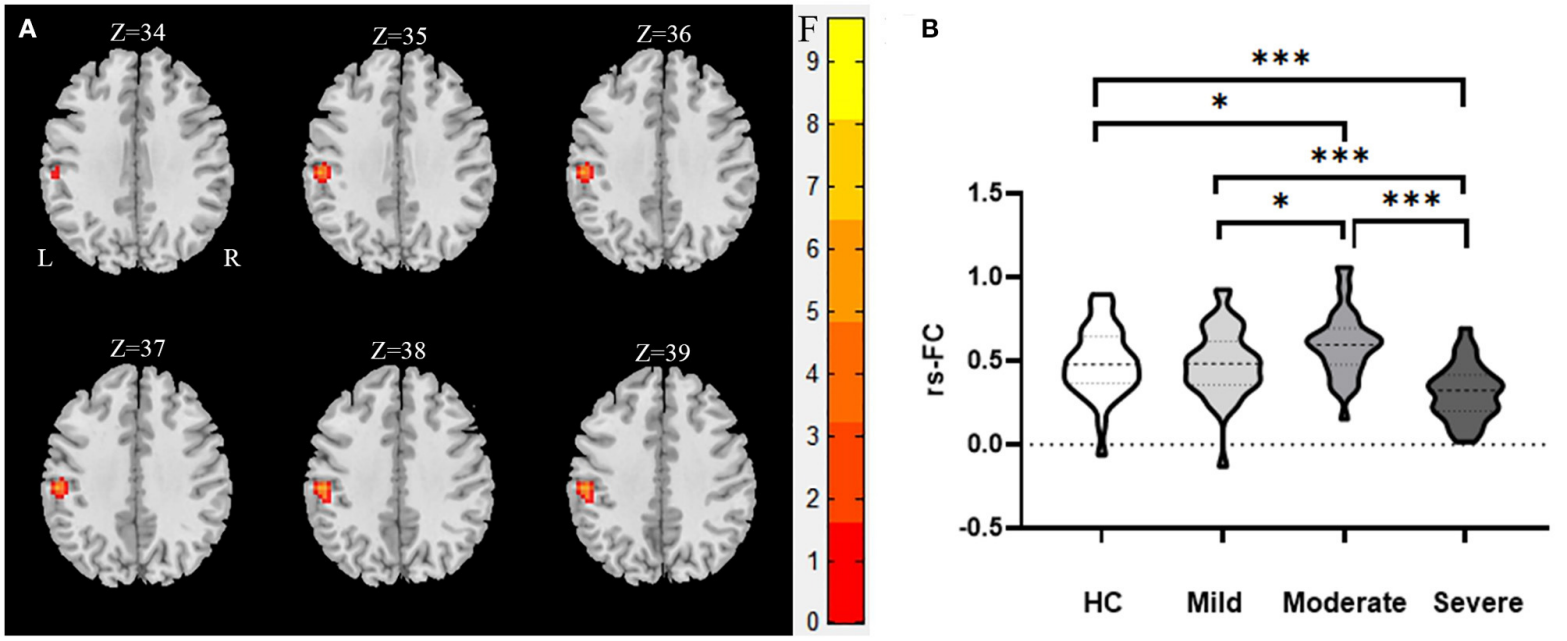
The abnormal functional connectivity between the left superior parietal gyrus and the left parietal inferior gyrus of the WMHs in MNI space. **(A)** Significant abnormal functional connectivities were observed between the left superior parietal gyrus and the left parietal inferior gyrus [34, 35, 36, 37, 38, 39 in **(A)** are the coordinates of Z-axis in Montreal Neurological Institute space, and the unit of coordinate system is millimeter]. **(B)** Mean values of the abnormal functional connectivities in these groups. ***Significant at 0.001 level and *significant at 0.05 level (2-tailed).

We used the right inferior frontal orbital gyrus and left superior parietal gyrus as seeds in the functional connectivity analysis of the whole brain. We found that the functional connectivity between the right inferior frontal orbital gyrus and left inferior temporal gyrus while and between the left superior parietal gyrus and the left parietal inferior gyrus differed significantly between the WMHs and HCs groups (Table 5; Figures 2, 3).

**Table 5:** Brain regions showing the changed functional connectivity in WMHs group.

**Brain regions**	**Peak MNI**	**Number of voxels**	***F*-value**	***P* (GRF)**
	**X Y Z**			
Left inferior temporal gyrus	−57, −60, −6	133	11.641	Cluster <0.05, voxel <0.001
Left parietal inferior gyrus	−54, −24, 39	131	9.6826	Cluster <0.05, voxel <0.001

*MNI, Montreal neurological institute; WMHs, white matter hyperintensities*.

### Correlation Analysis

Correlation analysis revealed a significant correlation between the DC value in the left superior parietal gyrus and MoCA score in HCs group (*P* = 0.033, *r* = −0.351) (Table 6).

**Table 6:** The correlation results between the DC value in the left superior parietal gyrus and the score of MoCA in HCs.

		**HCs-MoCA**	**WMHs-MoCA**	**HCs-left superior parietal**	**WMHs-left superior parietal**
HCs-MoCA	Pearson correlation	1	−0.027	−0.351	−0.274
	Significant (bilateral)		0.878	0.033^*^	0.101^*^
	*N*	37	35	37	37
WMHs-MoCA	Pearson correlation	−0.027	1	0.378	0.015
	Significant (bilateral)	0.878		0.025	0.879
	*N*	35	100	35	100
HCs-left superior parietal	Pearson correlation	−0.351	0.378	1	0.000
	Significant (bilateral)	0.033	0.025		0.998
	*N*	37	35	37	37
WMHs-left superior parietal	Pearson correlation	−0.274	0.015	0.000	1
	Significant (bilateral)	0.101	0.879	0.998	
	*N*	37	100	37	104

* *P < 0.05, HCs, healthy controls; WMHs, white matter hyperintensities*.

## Discussion

In this study, we combine data-driven DC analysis with ROI based FC. Firstly, we used DC analysis to describe the intrinsic abnormal functional connectivity of the whole brain functional network at the voxel level in patients with WMHs, and we found that patients with WMHs exhibited significant differences in DC values in the right inferior frontal orbital gyrus and left superior parietal gyrus compared to those in HCs. Then, we do further FC analysis, the further results indicated that the functional connectivity between the right inferior frontal orbital gyrus and left inferior temporal gyrus, left superior parietal gyrus, and left parietal inferior gyrus differed in patients with WMHs.

Previous studies have suggested that the right inferior orbital frontal gyrus is the upper part of the limbic lobe, and evidence has indicated that it is associated with a variety of brain functions, including memory-related emotions, self-awareness (35), cognitive regulation (36), memory, and reward (37). The right inferior frontal orbital gyrus is one of the core components of the default mode network (DMN) (38). Resting state activity is defined as the default mode of brain activity to denote a state in which an individual is awake and alert, but not actively involved in an attention-demanding or goal-directed task (39, 40). Since its discovery, interest has grown in the clinical utility and implications of the DMN (41, 42), and the clinical significance of the DMN has been established or implicated in neurological and neuropsychiatric disorders (40, 43, 44). A previous study showed that mild cognitive impairment (MCI) in Parkinson's disease is related to the disrupted connectivity in networks involved in cognition, primarily in the DMN (45). It was also reported that adolescent depression is associated with inflexibly elevated DMN connections (46). A previous study showed that the longitudinal trajectory of default mode network connectivity is associated with changes in episodic memory and processing speed (47). Furthermore, another study on children with temporal lobe epilepsy showed that there was a significant relationship between executive dysfunction and DMN (48). In recent years, some studies have pointed out that abnormal structural network connectivity is related to cognitive impairment in patients with WMHs (15, 16). Our results showed that the right orbital inferior frontal gyrus, an important part of DMN, whose DC value decreased, while the main cognitive impairment of WMHs are executive function and memory, which is consistent with previous studies. More recently, A meaningful study suggests that preferential destruction of cortical connections may lead to the progression of cognitive impairment in patients with WMHs (49), highlighting the role of reduced structural connectivity in the cognitive impairment of patients with WMHs. Previous studies have found that the left inferior temporal gyrus plays a critical role in working memory; consequently, impairment of the left inferior temporal gyrus leads to a decline in memory (50, 51), which is supported by the present study in some extent. We also found that the functional connectivity between the right inferior frontal orbital gyrus and left inferior temporal gyrus is significantly lower than that in the HCs group. Left inferior temporal gyrus, located on the lateral and inferior surface of the temporal neocortex, can be considered as a tertiary visual association cortex and the central portion of the language formulation area region, involving cognitive functions such as language, visual perception, and memory (52).

Previous studies have shown that in elderly people, executive function is supported by the left superior parietal (53, 54), and it has been suggested that the left superior parietal gyrus is engaged in basic attentional processing in executive function (55, 56). These suggestions were supported by our findings that patients with WMHs with lower DC values for the left superior parietal gyrus had poorer executive function. In addition, a previous study told us that the left superior parietal gyrus participates in cognitive control and integration (57). A decreased degree value indicates a decreased number of direct connections and reflects the decreased centrality or importance of a specific voxel in the brain network. In contrast to the right inferior frontal orbital gyrus, in the present study, we found that the DC value of the left superior parietal gyrus in the moderate WMHs group was significantly higher, which is consistent with a previous study, which suggested that the left superior parietal gyrus has a higher cortical thickness in patients with WMHs group than in HCs (58). In a voxel-based morphometry study, researchers observed an increase in gray matter density near thinning regions, indicating there is a compensatory mechanism in relevant brain regions (59). Furthermore, a regional increase in DC value might reflect an enhancement of function or compensatory hypertrophy in response to an acquired brain injury (i.e., WMHs). For example, changes in plasticity in individuals with an acquired brain injury most frequently leads to modifications in functional brain networks, which have been associated with distinct patterns of sensorimotor, behavioral, and cognitive impairments, are sparing (60). However, it is interesting to find an increase of DC values in the left superior parietal gyrus. Our study also found functional connectivity between the left superior parietal gyrus and left parietal inferior gyrus in the moderate WMHs group. If a potential compensatory mechanism could be confirmed in these regions, it could be used to help prevent WMHs-related degeneration. In conclusion, we found abnormal DC values in a variety of brain regions in the WMHs groups, which might demonstrate the reorganization of the brain network in response to WMHs.

In the present study, we found a significant negative correlation between the DC value in the left superior parietal gyrus and MoCA score in HCs. This indicates that a higher DC value is not necessarily better; on the contrary, exceeding a threshold will lead to a decrease in brain function. Human brain network has the property of small world. Too high may indicate wrong connectivity or invalid connectivity. The appearance of invalid connectivity or wrong connectivity will lead to functional damage. In previous studies, we can find that the left superior parietal gyrus is closely related to cognitive function. Similarly, we also found a negative correlation between the DC value of the left superior parietal gyrus and the overall cognitive MoCA score in the HCs group, which further tells us that there is a correlation between the left superior parietal gyrus and cognitive function. WMHs are mainly manifested as abnormal signals in white matter, which often indicates that the structural connectivities of white matter are changed, and the structural connectivities of white matter are the basis of functional connectivities. We speculated that WMHs can affect white matter connectivity and further cause abnormal functional connectivity of cortex and subcortical nuclei. In the present study, our results suggested that the DC value of the left superior parietal gyrus in WMHs groups were significantly changed, meanwhile, their cognitive function was also significantly different from that in HCs group, however, the correlation between DC value of left superior parietal gyrus and cognitive function in HCs group was not found in WMHs groups, which is consistent with our hypothesis that the white matter connectivity of patients with WMHs is changed, leading to changes in their related functional connectivity, leading to changes in cognitive function.

Since the clinical manifestations of WMHs are complex, we are unable to determine the mechanisms underlying these changes. Therefore, strict experimental design is crucial for future studies.

This study has a number of limitations. First, as a cross-sectional study, DC can only identify brain regions with abnormal functional connectivities and is unable to provide a clear causal relationship; we examined the functional connectivity based on defined ROIs. Second, the sample size was relatively small in the present study, which might have led to lower statistical power. Third, patients with WMHs were divided into three groups based on their Fazekas scores, which mainly emphasizes the average WMHs score but does not account for the actual brain areas with hyperintensities.

## Conclusion

In the present study, we used the DC analysis to explore the abnormal connectivity pattern in whole-brain functional networks in WMHs. We also correlated DC changes with MoCA scores. The current results showed decreased connectivity in the right inferior frontal orbital gyrus and changed connectivity in the left superior parietal gyrus. The functional connectivity between the right inferior frontal orbital gyrus and left inferior temporal gyrus was decreased, and the functional connectivity between the left superior parietal gyrus and left parietal inferior gyrus had changed. All in all, these results provide us a new approach to probe into complex WMHs. Taken together, the abnormal changes of DC values in brain regions may help us to better explore the underlying mechanism of cognitive function changes in WMHs, and this rs-fMRI study advanced our understanding of WMHs.

## Data Availability Statement

The datasets presented in this article are not readily available because The data that support the findings of this study are available on request the corresponding author. Requests to access the datasets should be directed to wangkai1946@126.com.

## Ethics Statement

The studies involving human participants were reviewed and approved by Anhui Medical University. The patients/participants provided their written informed consent to participate in this study.

## Author Contributions

BD: collect and analyze data and write articles. SC, CC, YL, XW, YM, and JN: collect data and statistical results. BQ: measurement services. QW: provide ideas and assisting in writing articles. PH and KW: guiding experimental ideas and assisting in writing articles. All authors contributed to the article and approved the submitted version.

## Conflict of Interest

The authors declare that the research was conducted in the absence of any commercial or financial relationships that could be construed as a potential conflict of interest.
